# Maternal, neonatal, pregnancy outcome characteristics of pregnant women with high plasma cell-free DNA concentration in non-invasive prenatal screening: a retrospective analysis

**DOI:** 10.3389/fped.2023.1195818

**Published:** 2023-08-17

**Authors:** Lingling Xing, Ting Bai, Sha Liu, Jianlong Liu, Xiaosha Jing, Cechuan Deng, Tianyu Xia, Yunyun Liu, Jing Cheng, Xiang Wei, Yuan Luo, Quanfang Zhou, Qian Zhu, Hongqian Liu

**Affiliations:** ^1^ Department of Obstetrics and Gynaecology, West China Second University Hospital, Sichuan University, Chengdu, China; ^2^ Key Laboratory of Birth Defects and Related Diseases of Women and Children, Ministry of Education, Sichuan University, Chengdu, China; ^3^ Department of Medical Genetics, West China Second University Hospital, Sichuan University, Chengdu, China

**Keywords:** non-invasive prenatal screening, cell-free DNA, systemic autoimmune disease, antiphospholipid syndrome, pregnancy complications, pregnancy-induced hypertension, pregnancy outcomes, preterm delivery

## Abstract

**Objective:**

Cell-free DNA (cfDNA) is a useful biomarker in various clinical contexts. Herein, we aimed to identify maternal characteristics and pregnancy outcomes associated with a failed NIPS test due to high cfDNA concentrations.

**Methods:**

A retrospective study of cases with high plasma cfDNA concentration in pregnant women in which NIPS test was performed (from 174,318 cases). We reported the detection of 126 cases (118 with complete clinical information) in which the high amount of cfDNA did not allow the performance of NIPS and study the possible causes of this result.

**Results:**

622 (0.35%) of 174,318 pregnant women had failed the NIPS test, including 126 (20.3%) cases with high plasma cfDNA concentrations. The failed NIPS due to high plasma cfDNA concentrations was associated with maternal diseases and treatment with low-molecular-weight heparin (LMWH). Further follow-up of the 118 pregnant women in the case group revealed that the pregnancy outcomes included 31 premature deliveries, 21 abortions. The cfDNA concentrations of pregnant women with preterm deliveries were 1.15 (0.89, 1.84), which differed significantly from those who had full-term deliveries.

**Conclusions:**

Among pregnant women with high cfDNA concentrations, systemic autoimmune diseases, pregnancy complications and LMWH were associated with increased incidence of failed NIPS test. High maternal cfDNA concentrations may not be associated with chromosomal abnormalities in the fetus. However, they should be alerted to the possibility of preterm births and stillbirths. Further clinical studies on pregnant women with high cfDNA concentrations are required.

## 1. Introduction

Serum, plasma, and other bodily fluids such as urine, cerebrospinal fluid, saliva or bronchial effusions are known to contain cell-free DNA (cfDNA), which is used as a valuable biomarker in different clinical contexts including prenatal diagnosis of genetic diseases, cancer detection, and phenotyping to select personalised treatments for cardiovascular and other diseases (1). Mandel and Metais were the first to report the presence of cfDNA in human plasma in 1948. In 1997, Lo et al. found free foetal DNA fragments in maternal plasma (2). It was later confirmed that approximately 10% of peripheral blood cfDNA in pregnant women is placental DNA (“foetal” DNA), and the remaining 90% is maternal cell DNA (3). Non-invasive prenatal screening (NIPS) was introduced into clinical practice in 2011 and quickly gained popularity within existing prenatal screening approaches. The method quantifies the risk of chromosomal abnormalities using foetal free DNA circulating in maternal blood. NIPS predominantly uses massively parallel sequencing to detect cell-free foetal DNA (cffDNA) fragments in maternal peripheral blood and a bioinformatics analysis of the sequencing results is performed to obtain important information. The size profile of cfDNA shares common features across populations and is distributed as a “ladder” pattern with a dominant peak at ∼166 bp. However, cfDNA demonstrates slight characteristic discrepancies owing to the origin of specific tissues. Dominant peaks of foetal and maternal cfDNA fragments in pregnant women are present at 143 and 166 bp (4). Foetal DNA in maternal circulation mainly originates from the placenta and foetal blood cells. Pregnancy increases total cfDNA in maternal circulation by ∼3%–13%, known as the “foetal fraction.” Maternal plasma cfDNA concentrations have been observed to markedly increase in patients with maternal diseases or complications (5). The aim of this study was to determine maternal characteristics and pregnancy outcomes related to high cfDNA concentrations and test failures to provide clinicians with reliable data and encourage pregnant women to undergo further clinical consultations.

## 2. Materials and methods

### 2.1. Study population and data collection

This was a retrospective study, conducted from April 2015 to December 2020, of pregnant women who underwent prenatal screening at the West China Second University Hospital of Sichuan University, Chengdu, Sichuan Province, China. We recorded baseline parameters such as age, height, weight, gestational age, medical history, and method of conception from the hospital's prenatal screening database. Data on pregnancy and neonatal outcomes were extracted from the hospital's electronic medical records and additional follow-ups. Our study case group consisted of 126 pregnant women who had failed NIPS quality control because of high maternal plasma cfDNA concentrations. Concurrently, our control group comprised 155,143 cases with qualified cfDNA quality control and complete clinical information. All participants who received the NIPS test have been conducted professional pre-test counselling and were fully aware of the application scope, target diseases, limitations, and possible inability to obtain results because of high or low cffDNA plasma concentration or alternative reasons. This study was approved by the Institutional Ethics Committee of Sichuan University, and all participants signed written informed consent prior to testing. We confirm that the research was performed in accordance with relevant clinical technical specifications.

### 2.2. Sample collection and testing

#### 2.2.1. NIPS

According to standard operating procedures, maternal peripheral blood (8–10 ml) was collected from all participants using Cell-free BCT Tubes (Streck, Omaha, NE, USA). Blood samples were left to stand at room temperature (<25°C) for 30 min and subsequently stored at 4°C. Collected blood samples were centrifuged for 10 min at 1,600 × *g* at 4°C within 72 h using an Eppendorf 5810R centrifuge (Eppendorf, Hamburg, Germany). Next, collected plasma was centrifuged for 10 min in an Eppendorf 5430R centrifuge at 16,000 × *g* at 4°C, and the remaining plasma was stored at −70°C. cfDNA was isolated from 1,200 µl plasma using a DNA extraction kit (Hangzhou Berry Gene Diagnostic Technology Co., Ltd., Hangzhou, China) following the manufacturer's instructions. The remaining plasma was stored at −70°C. The cfDNA concentration was measured using Qubit 3.0 and Ex-Kubit dsDNA HS test kits (Ex-Cell Biotech Co., Ltd., China). The normal cfDNA concentration range is 0.05–0.6 ng/µl; therefore, DNA was reextracted if the concentration fell outside of this qualifying range. If the concentration fell outside of the range a second time, it was deemed disqualified at the quality control stage, and the test was considered a failure.

#### 2.2.2. Invasive prenatal diagnosis

All pregnant women who failed the NIPS test received professional genetic counselling and were subsequently given the choice to undergo or reject invasive prenatal diagnostic procedures such as amniocentesis, cord blood puncture, or chorionic villus puncture. Diagnostic methods for pregnant women include copy number variation sequencing, chromosomal microarray analysis, and karyotype analysis.

#### 2.2.3. Pregnancy outcome follow-up

Of the 126 cases of NIPS failure, eight cases were lost to the follow-up; hence, the total follow-up results obtained amounted to 118 cases. First and second follow-ups were conducted 3–6 months after NIPS and 1 year post-partum, respectively. The average follow-up period was 533 days post-partum (or after pregnancy termination). Main follow-up evaluations included pregnancy complications, maternal diseases, drug use during pregnancy, pregnancy outcome, and neonatal growth and development. Third follow-ups were conducted 2 years post-partum, primarily for babies showing poor growth and development in the early follow-up.

#### 2.2.4. Statistical analyses

SPSS Version 21.0 software (IBM, Armonk, NY, USA) was used for data analyses. In this study, categorical and continuous variables with normal and skewed distribution were expressed as *N* (%), mean ± standard deviation (SD), and median (interquartile range, IQR). Comparisons between groups were determined using chi-square test, or Fisher's exact test, and Wilcoxon rank-sum test. Statistical significance was set at *P* < 0.05.

## 3. Results

**Figure 1:**
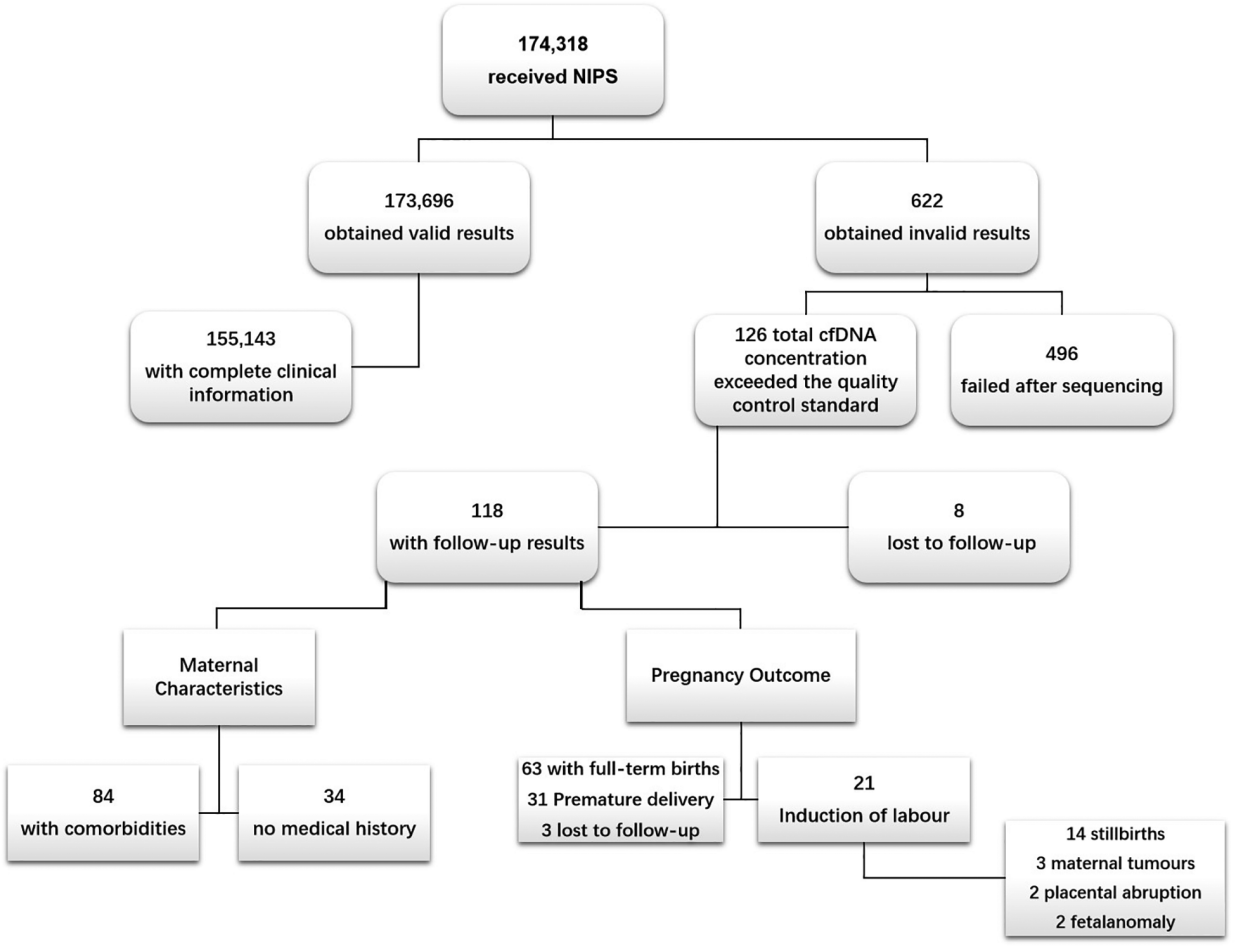
Flow chart of study population. NIPS, non-invasive prenatal screening. The causes of NIPS failure after sequencing were low foetal fraction (foetal fraction <4%) and sequencing that did not yield definitive results, i.e., sequencing failure.

Between April 2015 and December 2020, a total of 174,318 pregnant women of Chinese ethnicity received NIPS at the West China Second Hospital of Sichuan University, among which 622 cases failed to obtain valid results. The overall test failure rate was 0.35% (622/174,318). In these 622 cases, 126 cases failed because of high plasma cfDNA concentrations and were used as the case group. In the case group, 8 cases failed follow-up; 33 cases of amniocentesis showed no obvious abnormalities; 8 cases were re-tested at other institutions and showed normal results; further interventional prenatal diagnoses were not performed for the remaining 77 cases. In addition, 84 had comorbidities and 31 were premature. Meanwhile, 155,143 cases that received NIPS with qualified cfDNA concentrations and complete clinical information served as the control group (Figure 1) (Stable).

### 3.1. Characteristics of the research population

Demographic characteristics of the study population, including maternal age, gestational age, body mass index (BMI), disease, low-molecular-weight heparin (LMWH) usage, and ultrasound abnormalities are shown in Table 1. Maternal age differed significantly between case and control groups (*P* < 0.01), at 30.16 ± 4.86 (minimum: 19 years; maximum: 42 years) and 28.95 ± 4.81 (minimum: 14 years; maximum: 59 years), respectively. Prevalence rates of systemic autoimmune diseases (SAD), pregnancy-induced hypertension (PIH), tumours, and gestational diabetes were 42.1%, 15.8%, 4%, and 7.1%, respectively. In the case group, heparin was used in 31 individuals (24.6%), which differed significantly from that of the control group (*P* < 0.01) (Table 1).

**Table 1:** Population study descriptive statistics based on cfDNA concentration on NIPS (*n* = 155,269).

Characteristics	Controls: C <0.6	Cases: C ≥0.6	*P*
	*n* = 155,143 (%)	*n* = 126 (%)	
Maternal age (years)	28.95 ± 4.81	30.16 ± 4.86	0.005
Gestational age (weeks)	18 (16, 20)	18 (16, 20)	0.661
BMI (kg/m^2^)	22.19 (20.39, 24.24)	22.21 (20.02, 25.05)	0.929
SAD	87 (0.1)	53 (42.1)	<0.001
PIH (PE)	6 (<0.01)	20 (15.8)	<0.001
Tumour	49 (<0.01)	5 (4)	<0.001
GDM	12 (<0.01)	9 (7.1)	<0.001
LMWH	3 (<0.01)	31 (24.6)	<0.001
IVF	5,941 (3.8)	15 (11.9)	0.313
Foetal ultrasonography abnormalities	17,660 (11.4)	16 (12.7)	0.171

Data are expressed as median (P_25_, P_75_), mean ± SD, and *N* (%), Cases group: concentration of maternal plasma cfDNA ≥0.6 ng/µl; Control group: concentration of maternal plasma cfDNA <0.6 ng/µl. P_25_, P_75_, interquartile range; SD, standard deviation; cfDNA, cell-free DNA; NIPS, non-invasive prenatal screening; BMI, body mass index; GDM, gestational diabetes; PIH, pregnancy-induced hypertension; PE, preeclampsia; SAD, systemic autoimmune disease; IVF, *in vitro* fertilisation.

### 3.2. Maternal and new-born characteristics of individuals with high cfDNA concentration

**Figure 2:**
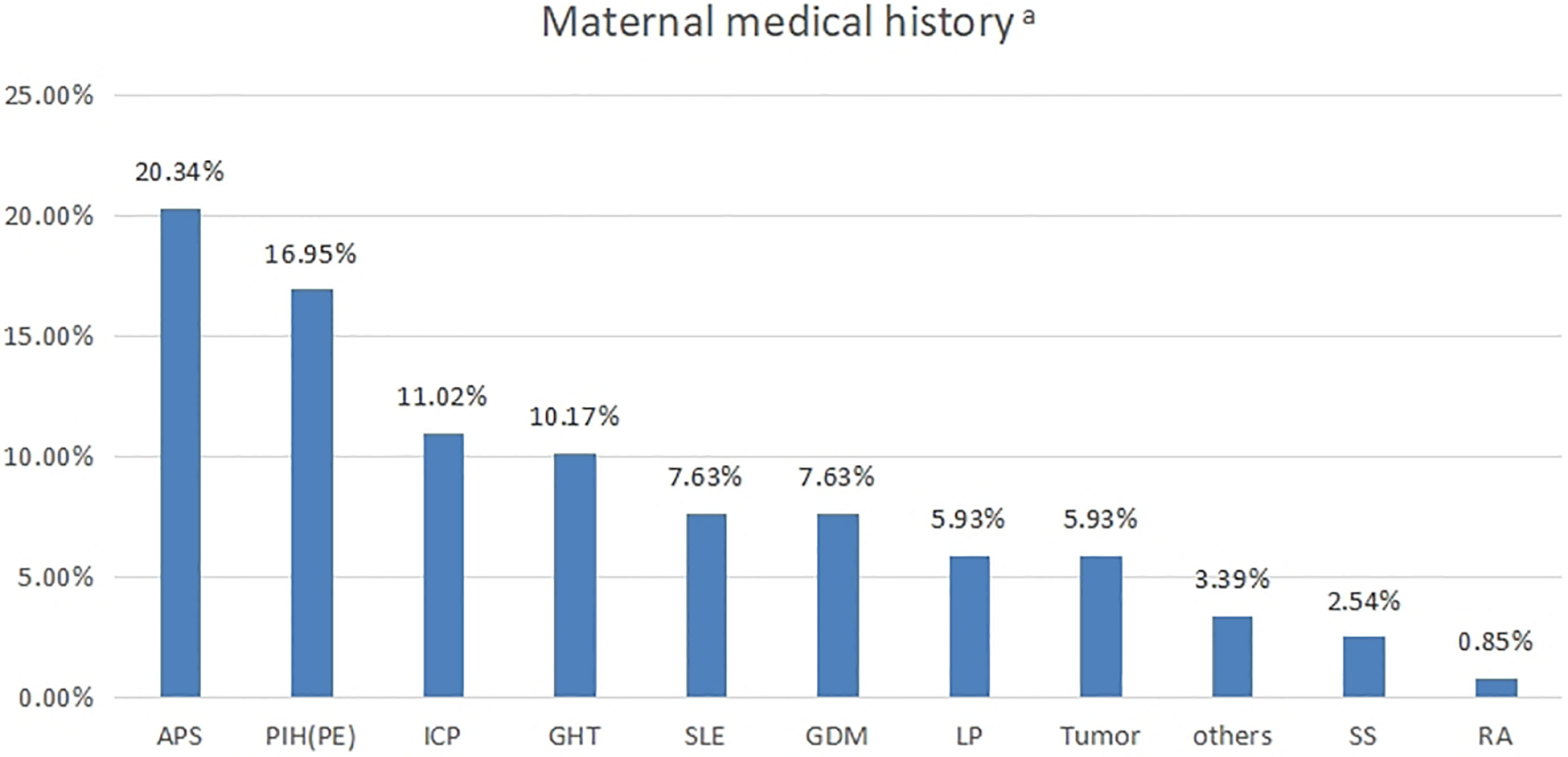
Proportion of maternal diseases (*n* = 118). ^a^Indicates 23 cases that developed two or more complications. APS, antiphospholipid syndrome; PIH, pregnancy-induced hypertension; PE, preeclampsia; ICP, intrahepatic cholestasis; GHT, gestational hypothyroidism; SLE, systemic lupus erythematosus; GDM, gestational diabetes; LP, thrombocytopenia; SS, Sjögren's syndrome.

The case group maternal diseases were primarily SAD as shown in Figure 2. SAD included antiphospholipid syndrome (APS), gestational hypothyroidism (GHT), thrombocytopenia (LP), systemic lupus erythematosus (SLE), and Sjögren's syndrome (SS). Maternal complications included PIH [including preeclampsia (PE)], intrahepatic cholestasis of pregnancy (ICP), and gestational diabetes mellitus (GDM). This grouping included multiple diseases and a single disease. Information on 23 cases with other mixed complications is shown in Supplementary Table.

Among the seven pregnant women with tumours in our study, three terminated their pregnancies because of acute myeloid leukaemia (AML), cerebral cancer, malignant mole, and PIH diagnoses in the second trimester. The patients with AML and cerebral cancer passed away due to their illness. Furthermore, we found no significant difference in cfDNA concentrations between pregnant women with multiple diseases and those with a single disease (Table 2).

**Table 2:** cfDNA concentration in NIPS based on case maternal medical history of multiple diseases and single diseases.

Medical history	M (P_25_, P_75_)	*P*
Single diseases	0.97 (0.79, 1.29)	0.149
Multiple diseases	1.12 (0.89, 1.84)	

In addition, Among individuals with high cfDNA concentrations, 49 had a history of spontaneous abortion. New-born height, weight, and BMI were 47.83 ± 3.43 cm, 2.59 ± 0.6 kg, and 11.66 ± 1.31, respectively. First and second follow ups identified 14 babies with developmental delay and two fetus with physical defects (one was induced due to ear deformity and the other was operated on after birth for abnormal genitalia). The third follow-up showed that previously stunted babies reached the Child Health Care standard value (Table 3).

**Table 3:** Overview of postpartum abnormalities.

Case number	Signs of dysplasia (after the first and second follow-up)	Prenatal ultrasound	Interventional prenatal diagnosis results	cfDNA	Maternal medical history	Maternal medication	Gestational weeks of delivery	Third follow-up status
S1	Growth retardation	(–)	Not tested	0.662	Nephrotic syndrome	(–)	FTP	Normal
S2	Growth retardation, Genital abnormality	abnormal S/D	Karyotyping: normal	11.7		LMWH	PTD	Normal
S3	Growth retardation	IUGR	Karyotyping: normal	0.743	SLE;SS	(–)	FTP	Normal
S4	Growth retardation	(–)	Not tested	1.33	LP	(–)	FTP	Normal
S5	Growth retardation	cholestasis	Not tested	0.724	ICP	uncertain	PTD	Normal
S6	Growth retardation	Abnormal Blood Flow Ratio of the Umbilical Artery and EICF	Not tested	1.84	GHT	Levothyroxine sodium	PTD	Normal
S7	Growth retardation	(–)	Not tested	1.01	SLE	(–)	FTP	Normal
S8	Growth retardation	(–)	Karyotyping: normal	0.925	Breast fibroma	traditional Chinese medicine	FTP	Normal
S9	Growth retardation	(–)	Not tested	1.09	PIH	(–)	PTD	Normal
S10	Growth retardation	(–)	Not tested	1.5	(–)	(–)	FTP	Normal
S11	Growth retardation	(–)	Not tested	2.28	APS;ICP;PIH	LMWH, Prednisone	FTP	Normal
S12	Growth retardation	(–)	CMA:normal	1.21	APS;ICP	LMWH etc.	FTP	Normal
S13	Growth retardation	(–)	CNV-seq:normal	1.004	APS	LMWH	FTP	Normal
S14	Growth retardation	(–)	CNV-seq:normal	0.616	SLE;GHT	LMWH etc.	FTP	Normal

EICF, echogenic intracardiac focus; IUGR, intrauterine growth restriction.

### 3.3. Maternal medication

Among the 118 cases, 45 were treated with medication during pregnancy. Medications mainly included LMWH (31, 68.9%), hormones, and levothyroxine sodium. Their cfDNA concentrations median (IQR) were 1 (0.84, 1.46), 0.99 (0.82, 1.28), and 0.91 (0.79, 1.23), respectively. And of all the pregnant women treated with medications, only one denied co-existing maternal disease.

### 3.4. Pregnancy outcome of high cfDNA concentration cases

Among the 118 cases, 3 had unknown outcome, 31 had premature births, 63 had full-term births, 21 had induced labour. Follow-up results of the 21 cases of pregnancies terminations (induction of labour) showed that 14 of them were due to stillbirth, 3 were due to acute myeloid leukemia (AML), cerebral cancer, and malignant mole in the second trimester, 2 were due to placental abruption, and the other 2 were due to cleft lip and palate, ear deformities of the foetal (Figure 1). In addition, maternal cfDNA concentrations of preterm pregnant women were significantly higher than those of full-term pregnant women (Table 4).

**Table 4:** Distribution of maternal cfDNA concentration under different pregnancy outcomes (*n* = 115).

Pregnancy outcomes	*N*	M (P_25_, P_75_)	*P*
FTP	63 (54.8%)	0.93 (0.76, 1.21)	0.04
PTD	31 (27%)	1.15 (0.89, 1.84)	

PTD, preterm delivery; FTP, full-term pregnancy.

Second follow-ups identified 14 cases with post-partum abnormalities. Six individuals underwent interventional prenatal diagnosis, which revealed normal results. In case S8, a lack of growth hormone was detected; however, their condition improved following medical rehabilitation. Case S2 was diagnosed with abnormal genitalia and underwent surgery. An additional follow-up found that previously self-reported abnormal growth and development parameters in children had returned to normal levels through post-correction (Table 3). In summary, the results of postpartum follow-up of 94 pregnant women indicated that the fetus had no significant chromosomal abnormalities.

## 4. Discussion

### 4.1. cfDNA and maternal diseases

Blood cfDNA levels are the result of the balance between cfDNA release and cfDNA clearance processes. In healthy individuals, cfDNA levels are low and rapidly cleared by cell apoptosis. Numerous studies have shown that serum or plasma levels of free circulating DNA (cDNA) are higher in cancer patients than in healthy individuals. Although plasma cfDNA levels are not recommended as a reliable screening tool, they have been documented to correlate with cancer stage and have prognostic value in predicting survival rate after chemotherapy or surgical treatment (6, 7). Among the seven pregnant women with tumours in our study, three terminated their pregnancy because of AML, cerebral cancer, malignant mole, and PIH diagnoses in the second trimester.

SADs are multifactorial disorders characterised by the appearance of autoreactive immune cells and specific autoantibodies. There are >100 human diseases classified as autoimmune or chronic inflammatory, which affect 5%–10% of the global population (8). In 1966, Tan et al. discovered high levels of cfDNA in patients with systemic lupus erythematosus (SLE) (9). Different mechanisms including apoptosis, necrosis, inflammatory response, or the release of active cells have been hypothesised as a source of cfDNA in SAD patients. In addition, the imbalance between DNA production and clearance has been postulated as an alternative mechanism (10). In our retrospective analysis of NIPS, dominant SADs in pregnant women with high cfDNA concentrations were APS (*n* = 24), GHT (*n* = 12), SLE (*n* = 9), LP (*n* = 7), and SS (*n* = 3). Most patients with APS had received subcutaneous LMWH injections immediately after pregnancy. Previous cffDNA quantitative analysis results of APS patients and healthy subjects have not shown significant differences (11). However, our retrospective analysis identified 24 APS patients in the high cfDNA concentration case group, the largest number of all confirmed SADs, so cfDNA in pregnant women may predominantly originate from maternal cells rather than foetal cells. In addition, women with hypothyroidism may experience pregnancy complications, including high blood pressure and PE. In our study, among the 12 patients with GHT, 6 had multiple other complications. It is uncertain whether GHT is associated with increased cfDNA concentrations. Notably, Hirata et al. studied differentially expressed genes in the circulating RNA of pregnant women with TSH levels marginally higher than the normal range and found that high levels of circulating RNA could be detected, even in women with mildly elevated TSH (12). SLE is an autoimmune disease that causes damage in parts of the body, including skin, kidneys, and joints and is characterised by the production of antibodies against nuclear antigens known as “anti-nuclear antibodies.” Recent research has shown that compared with healthy controls, SLE patients have higher cfDNA levels. In addition, compared with patients in the inactive phase, SLE patients in the active phase of the disease have higher cfDNA levels. This suggests that cfDNA levels may be a potential tool for assessing and predicting disease activity in SLE patients (13). Furthermore, SS patients have shown high cfDNA blood serum levels, with a significant correlation between cfDNA concentrations and disease activity (14). Notably, one of the three pregnant women with SS in our study suffered from hypothyroidism and LP. Among the seven pregnant women with LP discovered at the time of NIPS, three were diagnosed with thrombocytopenic purpura. Sui et al. found that compared with healthy controls, the cfDNA blood plasma concentration of patients with ITP significantly increased. Thus, as one of the results of ITP, cfDNA concentration may help stratify patients with a high mortality risk during acute immune thrombotic thrombocytopenic purpura episodes (15).

### 4.2. Maternal medication

As shown in Table 1, our findings reveal that LMWH may affect cfDNA concentrations in the maternal peripheral blood plasma of pregnant women undergoing NIPS, which was in line with previous results (16). LMWH has shown a strong affinity for DNA polymerase and affected Mg^2+^ concentrations in PCR assays, and the plasma DNA in pregnant women has been suggested to contain a higher proportion of small DNA fragments with abnormally high levels of guanosine–cytosine content (17). However, some scholars have stated that these studies lack appropriate methodology and provide insufficient evidence, some maternal factors may have caused cfDNA concentration to increase, heparin treatment had no effect on cfDNA screening for aneuploidy and that SAD presence was an independent predictor of invalid results (18). Meanwhile, further studies by Shree et al. showed that, in the absence of autoimmune disease, anticoagulation use, but not aspirin, is associated with lower FF, higher total cfDNA concentration, and higher rates of indeterminate results. Anticoagulation use was not accompanied by differences in cfDNA fragment size or GC content. Statistical differences in chromosome level *Z*-scores did not clinically affect aneuploidy detection (19).

### 4.3. Pregnancy complications

PE patients have demonstrated increases in cfDNA levels before the onset of PE and cffDNA concentrations after the onset of symptoms, which increased 5-fold compared to that of healthy individuals. Further, it has been found that foetal and maternal cfDNA concentration increases according to disease severity (20–23). As of the last follow-up, the babies of nine women with GDM had normal growth and development. GDM is a transient abnormal glucose intolerance during pregnancy that is diagnosed in the late second or early third trimester. All pregnant women are screened for GDM in the second trimester with protocols varying between countries. Therefore, results to date are conflicting, and more investigations are needed to clarify the use of cfDNA as a possible risk factor for GDM (5). Furthermore, maternal age may have an important role in the association between plasma cfDNA levels and GDM (24). The most common liver disease experienced during pregnancy is ICP, which occurs in the third trimester and subsides quickly after delivery. It is characterised by itching and elevated serum bile acid concentrations. Of the 13 pregnant women with ICP, only two had full-term delivery; the remaining 11 delivered prematurely. All women who gave birth at full-term received LMWH injections in the first trimester. Notably, Yi et al. found that women affected by ICP had higher cffDNA values (25). In addition, Yuan et al. investigated the association between the cfDNA levels measured during NIPS testing and the risk of pregnancy complications in the Chinese population and found that the increase in cfDNA levels is related to the increased risk of ICP (24). The association between increased concentration of cfDNA in pregnant women with ICP-related increase in placental oxidative stress and apoptosis was investigated by Perez et al. They found that in obstructive cholestasis during pregnancy (OCP) in rats, there were signs of placental oxidative stress (lipid peroxidation and protein carbonylation). Further, real-time quantitative PCR revealed that OCP induced an increase in Bax-a/Bcl-2 mRNA ratio, suggesting enhanced susceptibility to apoptosis activation through the mitochondria-mediated pathway (26).

### 4.4. cfDNA and pregnancy outcome

We found significant differences in cfDNA levels between pregnant women that delivered pre- and full-term babies (Table 4). Carbone et al. postulated the following two main hypotheses regarding the association between PTD and cfDNA concentration: (1) a higher cfDNA value could be related to the breakdown of the placental barrier in line with labour onset and/or with preliminary events that precede labour; however, this mechanism appears to be more related to labour itself and not PTD; (2) cffDNA being non-self DNA could play a pro-inflammatory role that activates several factors, including the Toll-like receptor (TLR) 9 pathway and stimulator of interferon genes (STING) pathway. Activation of the TLR9 pathway results in the production of pro-inflammatory cytokines and STING pathway activation results in the release of interferon-β and interferon-α and other pro-inflammatory mediators. Thus, it is unclear whether elevated cfDNA is a consequence or a cause of PTD (5). Stillbirth is one of the most common adverse pregnancy outcomes. In our study, 14 pregnancies with high cfDNA were terminated due to stillbirth. The presence of maternal diseases increases the likelihood of developing a stillbirth during pregnancy or delivery, including: diabetes mellitus, pregnancy complications and intrauterine growth restriction were associated with stillbirth. Pregnancy with Rhesus isoimmunisation, pregnancy-induced hypertension and gestational diabetes also had higher odds of stillbirth (27).

Although our second follow-ups identified 14 individuals with post-partum abnormalities (Table 3), regarding pregnant women with NIPS test failures owing to high cfDNA concentrations, existing data cannot prove that foetal growth restriction was related to cfDNA concentration. As most women suffered from other diseases during pregnancy, we cannot say with certainty whether growth retardation was a consequence of pregnancy complications. Sekizawa et al. reported that there was no increase in foetal DNA in the plasma of pregnant women with foetal growth restriction (28). Moreover, in those 14 fetuses, those who received prenatal diagnosis had normal chromosomal results; in those who did not receive a diagnosis, follow-up interviews also showed normal results. High maternal cfDNA concentrations may not be associated with chromosomal abnormalities in the fetus.

## 5. Conclusions

Our research provides some evidence that high cfDNA concentrations may be associated with SAD (APS, GHT, SLE, LP, and SS), pregnancy complications (PIH/PE, GDM, and ICP) and premature delivery, but not with foetal-specific factors. High cfDNA concentrations in pregnant women may not be associated with chromosomal abnormalities in the fetus. Thus, pregnant women who failed NIPS testing solely because of high cfDNA concentrations do not necessarily require invasive prenatal diagnosis; But they should be alerted to the possibility of preterm birth and stillbirths; Whether to choose invasive prenatal diagnosis or other screenings can be considered in combination with the pregnant woman's personal wishes and other inspection indicators; Meanwhile, pregnant women who have been diagnosed with related diseases (APS, PIH, ICP, etc.) or are not eligible for NIPS should be informed that NIPS may fail. Further, concerning NIPS testing, the clinical significance of elevated cfDNA concentration in pregnant women still requires further exploration, it is unclear if quantitative maternal cfDNA concentration can be used to predict diseases. It is particularly important to undergo effective genetic counselling before receiving NIPS, so that pregnant women who have been diagnosed with related diseases mentioned above or have used LMWH may receive results accordingly. Owing to heterogeneity and difficulty interpreting publicly available data, it is not yet possible to draw conclusions about statistical and clinical relevance. Therefore, other methods should be used for clinical verification for pregnant women who fail the NIPS test because of high cfDNA concentration while considering related diseases.

## Data Availability

The original contributions presented in the study are included in the article/**Supplementary Material**, further inquiries can be directed to the corresponding authors.
